# Activation of AKT by hypoxia: a potential target for hypoxic tumors of the head and neck

**DOI:** 10.1186/1471-2407-12-463

**Published:** 2012-10-10

**Authors:** Hanneke Stegeman, Johannes H Kaanders, Deric L Wheeler, Albert J van der Kogel, Marieke M Verheijen, Stijn J Waaijer, Mari Iida, Reidar Grénman, Paul N Span, Johan Bussink

**Affiliations:** 1 Department of Radiation Oncology, Radboud University Nijmegen Medical Centre, PO Box 9101, 6500 HB, Nijmegen, The Netherlands; 2 Department of Human Oncology, University of Wisconsin School of Medicine and Public Health, 1111 Highland Ave, Madison, WI 53705, USA; 3 Department of Otorhinolaryngology-Head and Neck Surgery and Department of Medical Biochemistry, Turku University Hospital and University of Turku, PO Box 52, FI-20521, Turku, Finland

**Keywords:** Head and neck cancer, Tumor microenvironment, Hypoxia, pAKT, EGFR

## Abstract

**Background:**

Only a minority of cancer patients benefits from the combination of EGFR-inhibition and radiotherapy in head and neck squamous cell carcinoma (HNSCC). A potential resistance mechanism is activation of EGFR and/or downstream pathways by stimuli in the microenvironment. The aim of this study was to find molecular targets induced by the microenvironment by determining the *in vitro* and *in vivo* expression of proteins of the EGFR-signaling network in 6 HNSCC lines. As hypoxia is an important microenvironmental parameter associated with poor outcome in solid tumors after radiotherapy, we investigated the relationship with hypoxia *in vitro* and *in vivo*.

**Methods:**

Six human HNSCC cell lines were both cultured as cell lines (*in vitro*) and grown as xenograft tumors (*in vivo)*. Expression levels were determined via western blot analysis and localization of markers was assessed via immunofluorescent staining. To determine the effect of hypoxia and pAKT-inhibition on cell survival, cells were incubated at 0.5% O_2_ and treated with MK-2206.

**Results:**

We observed strong *in vitro*-*in vivo* correlations for EGFR, pEGFR and HER2 (r_s_=0.77, p=0.10, r_s_=0.89, p=0.03) and r_s_=0.93, p=0.02, respectively), but not for pAKT, pERK1/2 or pSTAT3 (all r_s_<0.55 and p>0.30). *In vivo,* pAKT expression was present in hypoxic cells and pAKT and hypoxia were significantly correlated (r_s_=0.51, p=0.04). We confirmed *in vitro* that hypoxia induces activation of AKT. Further, pAKT-inhibition via MK-2206 caused a significant decrease in survival in hypoxic cells (p<0.01), but not in normoxic cells.

**Conclusions:**

These data suggest that (p)EGFR and HER2 expression is mostly determined by intrinsic features of the tumor cell, while the activation of downstream kinases is highly influenced by the tumor microenvironment. We show that hypoxia induces activation of AKT both *in vitro* and *in vivo,* and that hypoxic cells can be specifically targeted by pAKT-inhibition. Targeting pAKT is thus a potential way to overcome therapy resistance induced by hypoxia and improve patient outcome.

## Background

Head and neck squamous cell carcinoma (HNSCC) is the sixth most common cancer with 500.000 new diagnoses per year worldwide [1]. Recent research findings have resulted in a better understanding of the biologic features of HNSCC tumors, which has led to the development of new therapeutic agents targeting specific molecules important for tumor growth and cell survival. One of these new successful targeting agents is Cetuximab, a monoclonal antibody against the Epidermal Growth Factor Receptor (EGFR), which improves survival in HNSCC patients treated with radiotherapy [2]. However, despite this success, a significant proportion of patients does not benefit from the addition of anti-EGFR treatment. In addition, most clinical trials find no correlation between EGFR expression assessed by immunohistochemistry and response to treatment with EGFR inhibitors [3].

To improve patient outcome with these new intensified treatments, a large amount of research has been focused on identifying resistance mechanisms using different *in vitro* models [4-7]. However, it is uncertain to what extent these *in vitro* results can be translated towards the *in vivo* situation. A potential reason for the absence of a correlation between EGFR expression and response to EGFR inhibition is the EGFR-independent activation of signaling pathways, including the phosphatidylinositol-3-kinase (PI3-K)/protein kinase B (AKT) pathway, by other stimuli in the microenvironment [8]. The tumor microenvironment is complex and includes fluctuating oxygen and nutrient gradients, which are not present in standard 2D cell culture assays, but which can have a great impact on tumor behavior and treatment response. Hypoxia is an important microenvironmental parameter known to induce the transcription and activation of a wide variety of proteins and is an inherent negative factor for treatment outcome in solid tumors, including treatment outcome after radiotherapy [9-11]. Hence, it is of great importance to discover molecular targets that could be used to specifically kill hypoxic cells and consequently improve patient outcome.

Therefore, the aim of this study was to find molecular targets in the EGFR-pathway, which are induced by the microenvironment and thus not detected in standard *in vitro* assays. For this, we determined the *in vitro* and *in vivo* expression of proteins involved in the EGFR-signaling network in HNSCC lines both cultured as cell lines and grown as xenograft tumors. We investigated the tyrosine kinase receptors EGFR and human epidermal growth factor receptor 2 (HER2), and the activated form of kinases of the main signaling pathways downstream of EGFR: protein kinase B (AKT), extracellular signal-regulated kinase 1/2 (ERK1/2), and signal transducer and activator of transcription 3 (STAT3) [12]. Using this method, we were able to determine that pAKT, pERK1/2 and pSTAT3 were differentially expressed between cells in culture and in tumors, as an indication that these proteins were likely to be influenced by the tumor microenvironment. More importantly, we observed that AKT is activated by hypoxia both *in vivo* and *in vitro* and that the hypoxic cells are more sensitive to AKT-inhibition. These data implicate that pAKT-inhibition could be a promising way to specifically target hypoxic tumors in the clinic and improve outcome after a variety of treatments, including radiotherapy and EGFR-inhibition.

## Methods

### Cell lines

Six human head and neck squamous cell carcinoma cell lines (UT-SCC lines) were both cultured *in vitro* and grown as xenografts in nude mice. The characteristics of the cell lines are shown in Table 1. Cells were cultured in T75 culture flasks, under humidified conditions (37°C, 5% CO_2_), and passaged weekly or twice weekly in DMEM containing 2 mM L-glutamine, 1% nonessential amino acids, 20 mM Hepes, 10 units/ml penicillin, 10 units/ml streptomycin, and 10% fetal bovine serum.

**Table 1:** Characteristics of UT-SCC cell lines

**Cell line**	TNM*	**Primary tumor location**	**Type of lesion**	**Grade**
UT-SCC5	T_1_N_1_M_0_	Tongue	Primary	2
UT-SCC8	T_2_N_0_M_0_	Supraglottic larynx	Primary	1
UT-SCC15	T_1_N_0_M_0_	Tongue	Recurrence	1
UT-SCC29	T_2_N_0_M_0_	Glottic larynx	Primary	1
UT-SCC38	T_2_N_0_M_0_	Glottic larynx	Primary	2
UT-SCC45	T_3_N_1_M_0_	Floor of mouth	Primary	3

*TNM status of primary tumors according to the International Union against Cancer (1997).

Note: Grade: 1, well differentiated; 2, moderately differentiated; 3, poorly differentiated.

### Hypoxic incubation and MK-2206 treatment

To determine expression levels after hypoxia and/or AKT-inhibition, UT-SCC5 and UT-SCC15 cells were treated overnight (16h) with 0 or 2 μM MK-2206 (Selleckchem, Houston, TX, USA) under standard normoxic conditions and thereafter incubated under normoxic conditions or under hypoxic conditions (0.5% O_2_, H35 hypoxystation, Don Whitley Scientific Ltd., West Yorkshire, UK) for 1h.

To assess cell survival after hypoxia and/or AKT-inhibition, UT-SCC5 and UT-SCC15 cells were seeded in 96-well plates. After the cells were allowed to attach overnight under standard normoxic conditions, 0 or 2 μM MK-2206 was added and cells were incubated under normoxic conditions or under hypoxic conditions (0.5% O_2_) for 72h. Cell survival was determined 72h after normoxic or hypoxic incubation using a Cell-Counting Kit-8 assay (CCK8, Sigma-Aldrich Chemie BV, Zwijndrecht, The Netherlands).

### Xenograft tumors

Animal experiments were performed using 6–8 week-old BALB/c nu/nu mice. Of all six carcinoma cell lines, 5x10^6^ cells were injected subcutaneously into the flank. Tumor size was measured by the same technician twice a week. Tumors with a diameter of 4 mm or larger were harvested or passaged. For passaging, the tumor was excised and cut into 1 mm^3^ tumor pieces. The tumor pieces were then subcutaneously implanted into the flank. In this study first, second, and third passage tumors were analyzed. One hour before tumor excision, animals were injected intraperitonally with 0.5 ml of saline containing 2 mg pimonidazole hydrochloride (1-[(2-hydroxy-3-piperidinyl) propyl]-2-nitroimidazole hydrochloride, Natural Pharmaceuticals International Inc., Research Triangle Park, NC, USA) to label hypoxic cells. After excision, tumors were immediately frozen in liquid nitrogen. The number of harvested tumors ranged from 2 to 4 per cell line with a total of 19.

Animals were kept in a specific pathogen-free unit in accordance with institutional guidelines. All experiments were approved by the Animal Experiments Committee of the Radboud University Nijmegen Medical Centre.

### Western blot analysis

To determine protein expression *in vitro* and *in vivo*, cultured cells or tumor sections were lysed, cell debris was removed by centrifugation and protein was quantitated using a standard Bradford absorbance assay. Proteins (25 μg per lane) were separated by SDS-PAGE and blotted onto PVDF membrane. Membranes were incubated with the appropriate primary antibodies followed by incubation with HRP-conjugated antibodies. Finally, proteins were detected with an ECL chemiluminescence system. Antibodies against the following antigens were used: EGFR, pEGFR (Y1173), HER2, AKT, STAT3, and HRP-conjugated goat-anti-rabbit IgG, goat-anti-mouse IgG and donkey-anti-goat IgG were purchased from Santa Cruz Biotechnology Inc. (Santa Cruz, CA, USA). pHER2(Y1221/1222), pAKT(S473), pERK1/2(T202/Y204), ERK1/2, and pSTAT3(Y705) were purchased from Cell Signaling Technology (Beverly, MA, USA) and α-tubulin was obtained from Calbiochem (San Diego, CA, USA).

To obtain a quantitative measure for total protein expression, the integrated optical density (IOD) of the chemiluminescent signal was measured using ImageJ software (NIH, Bethesda, MD, USA). IOD values of all proteins were normalized to those of α-tubulin.

### Immunohistochemical staining, image acquisition and analysis of tumor sections

To determine localization of protein expression *in vivo*, frozen tumor sections (5 μm) were thawed, fixed in acetone (4°C) and rehydrated in PBS. Two consecutive tumor sections from each tumor were stained for pAKT or pimonidazole in combination with EGFR and vessels. EGFR and pAKT were stained with the same antibodies used for western blot analysis. The antibody against pimonidazole was a gift from J.A. Raleigh (University of North Carolina) and 9F1 supernatant, a rat monoclonal antibody against mouse endothelium, was a gift from the Department of Pathology, Radboud University Nijmegen Medical Centre. EGFR was detected by incubation with Cy3-conjugated donkey-anti-goat IgG (Jackson Immuno Research Laboratories Inc., West Grove, PA, USA), pAKT and pimonidazole by incubation with Alexa488-conjugated donkey-anti-rabbit IgG (Molecular Probes, Leiden, The Netherlands), and 9F1 by incubation with Alexa647-conjugated chicken-anti-rat IgG (Molecular Probes). Stained sections were mounted in Fluorostab (ICN Pharmaceuticals, Inc, Zoetermeer, The Netherlands).

Stained tumor sections were scanned on a digital image processing system consisting of a 12-bit charge-couple device camera (Micromax, Roper Scientific Inc., Trenton, NJ, USA) on a fluorescence microscope (Axioskop, Zeiss Göttingen, Germany) and a computer-controlled motorized stepping stage, using IPLab software. Each section was sequentially scanned three times at 100x magnification, yielding an image of hypoxia (pimonidazole) or pAKT-expression, an image of EGFR-expression and an image of the vasculature structures (9F1). One pseudo-colored composite image was reconstructed from the individual microscope images. Using this composite image, total tumor area was delineated and non-tumor tissue, necrotic area and staining artifacts were excluded from the analysis. Thereafter, thresholds for the fluorescence signals were interactively set at intensities where the steepest gradient occurred between background and foreground intensity levels, and grey value images were converted to binary images. Using ImageJ software (NIH, Bethesda, MD, USA), the fraction positive for pAKT or hypoxia was calculated by dividing the tumor area positive for the respective marker by the total tumor area. The overlap fraction of EGFR and pAKT was calculated by dividing the tumor area positive for both markers by the tumor area positive for EGFR.

### Statistics

Statistical analyses were performed using Prism 4.0c (GraphPad Software, Inc., LA Jolla, CA, USA). Correlations between parameters were assessed using the Spearman correlation test. The significance of differences in cell survival between different treatments was assessed using a Kruskal-Wallis test in combination with a Dunn’s multiple comparison test. P-values < 0.05 were considered to be significant.

## Results

### Expression of activated kinases, but not of tyrosine kinase receptors, induced by tumor microenvironment

**Figure 1:**
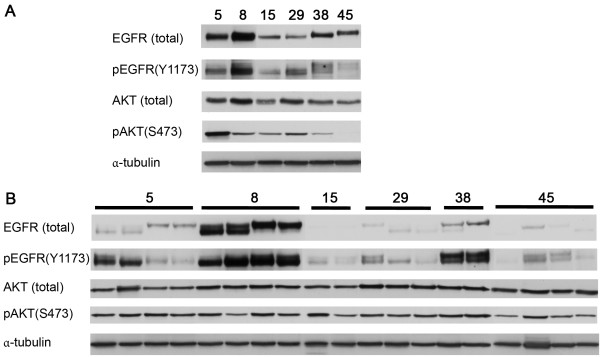
***In vitro *and *in vivo *expression of EGFR, pEGFR, pAKT and AKT in 6 HNSCC lines.** Cell lines were both cultured as cell lines (*in vitro*) and grown as xenograft tumors (*in vivo*) and expression levels were determined with western blot. Expression of α-tubulin was used as loading control. **A**) *In vitro* expression of EGFR, pEGFR, pAKT and AKT. **B**) *In vivo* expression of EGFR, pEGFR, pAKT and AKT. Number of harvested tumors ranged from 2 to 4 per cell line.

Using western blot analyses, expression levels of various proteins involved in the EGFR-signaling network were assessed in 6 different HNSCC cell lines both grown in culture (*in vitro*) and grown as xenograft tumors (*in vivo*). As example, western blots for (p)EGFR and pAKT are shown in Figure 1 (western blot images of (p)HER2, (p)ERK1/2 and (p)STAT3 are shown in Additional file 1: Figure S1).

**Figure 2:**
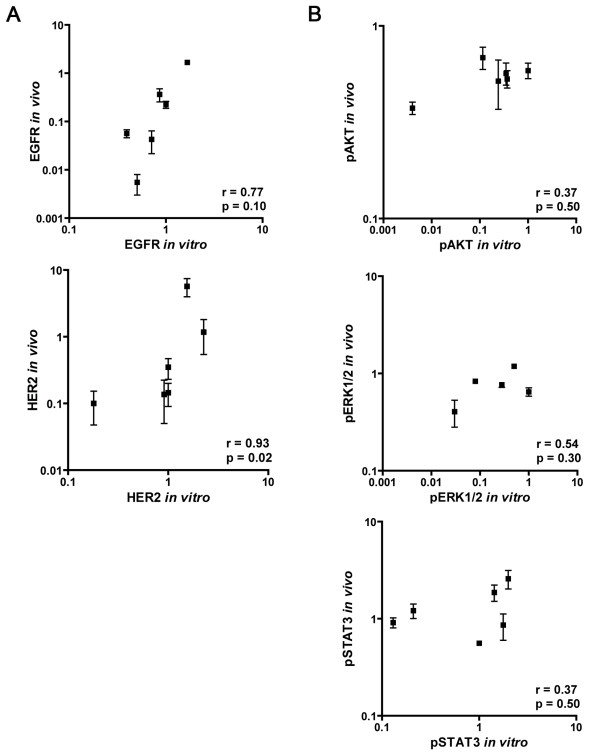
**Correlation between *in vitro *and *in vivo *expression of EGFR/HER2 and pAKT/pSTAT3/pERK1/2 in 6 HNSCC lines.A**) Correlation between *in vitro* and *in vivo* expression of EGFR and HER2. **B**) Correlation between *in vitro* and *in vivo* expression of pAKT, pERK1/2 and pSTAT3. Expression was assessed by western blot analysis and depicted in relative units. The integrated optical density (IOD) was measured and all values were normalized to those of α-tubulin by dividing the IOD value for that specific marker by the IOD value of α-tubulin. *In vitro* expression of UT-SCC5 was set as standard. Error bars represent standard error of the mean and all axes are in log scale. Correlations between *in vitro* and *in vivo* expression were assessed using the Spearman correlation test.

The tyrosine kinase receptors EGFR and HER2 showed a wide variation of expression levels in both cells and tumors. Overexpression of these membrane receptors seems to be an intrinsic feature of the cell lines as for both EGFR and HER2 a strong correlation was found between *in vitro* and *in vivo* expression levels (r_s_=0.77, p=0.10 and r_s_=0.93, p=0.02, respectively) (Figure 2A). Although there was only a trend towards a correlation between *in vitro* and *in vivo* EGFR expression (p=0.10), the correlation for the activated form pEGFR was significant (r_s_=0.89, p=0.03). This was not the case for pHER2 (r_s_=0.26, p=0.66), which showed different levels of expression in the tumors, but was invariably low *in vitro*.

In contrast to the tyrosine kinase receptors, no *in vitro-in vivo* correlation was observed for the activated kinases pAKT, pERK1/2 or pSTAT3 (all r_s_<0.55 and p>0.30) (Figure 2B). The lack of correlation was predominantly due to the fact that there was a wide range of expression levels *in vitro* while *in vivo* the kinases were activated at relatively high levels in all tumor lines (Figure 1 and 2B). These results suggest that stimuli in the tumor microenvironment activate the different cell signaling pathways and consequently lead to relatively high *in vivo* levels of pAKT, pERK1/2 and pSTAT3.

### pAKT expression present in hypoxic cells in vivo

A variety of stimuli within the tumor microenvironment can activate kinases like AKT, ERK1/2 and STAT3, including hypoxia. Therefore, EGFR, pAKT, and hypoxia were immunohistochemically stained in tumor sections to visualize their spatial relationship and to find a possible explanation for the absence of a correlation between the *in vitro* and *in vivo* expression of pAKT.

**Figure 3:**
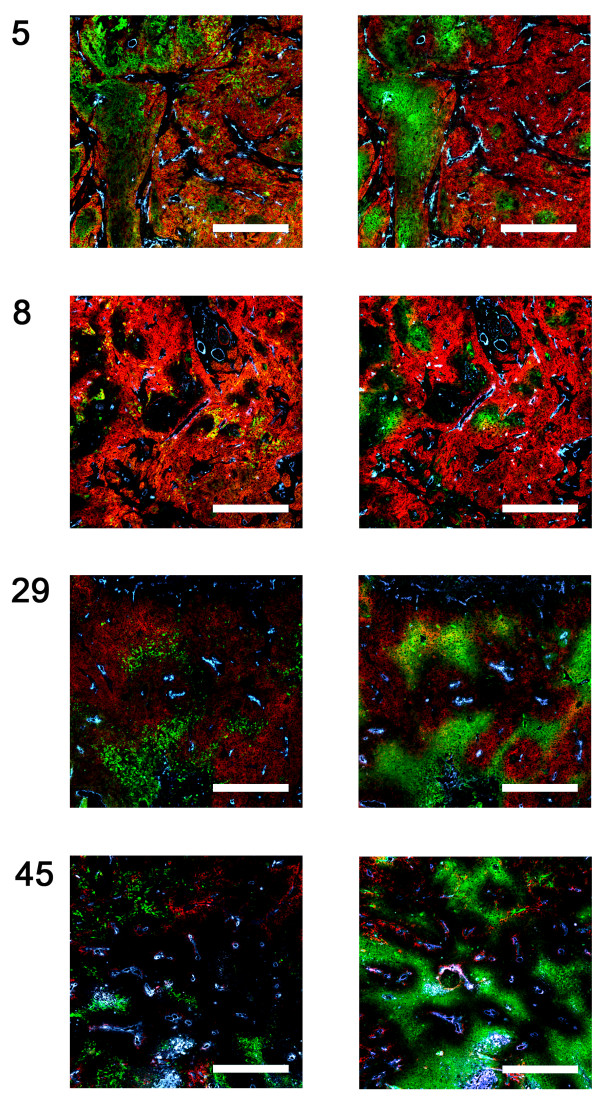
**Expression of EGFR, pAKT and hypoxia *in vivo*.** Expression of EGFR and pAKT in relation to hypoxia was analyzed by immunohistochemical analysis in UT-SCC xenograft tumors of 4 different lines. Left column: EGFR (red), pAKT (green), vessels (blue). Right column: EGFR (red), hypoxia (green), vessels (blue). Non-specific staining present in necrotic regions. Scale bars represent 500 μm. Magnification: 100X.

**Figure 4:**
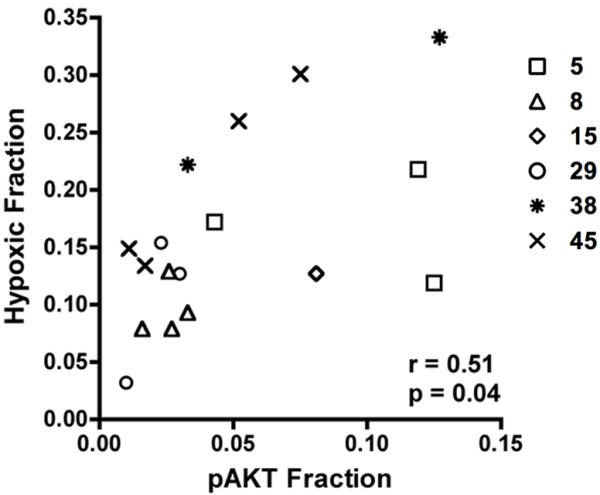
**Correlation between pAKT and hypoxia *in vivo *.** Correlation between the pAKT and hypoxic fraction in tumors of 6 HNSCC lines was assessed with the Spearman correlation test. Symbols represent individual tumors of the different UT-SCC lines.

We observed that EGFR expression was predominantly present in tissue surrounding blood vessels, whereas pAKT was mostly expressed further away from the vessels in hypoxic areas indicated by pimonidazole staining in the consecutive tumor section (Figure 3 and Additional file 2: Figure S2). Using quantitative image analysis, we observed that in average only 7.7% (range: 1.0-33.6%) of cells expressing EGFR also expressed pAKT. Thus, although cells were present that expressed both EGFR and pAKT, the overall overlap was relatively low. Moreover, we found that the fraction of the tumor positive for pimonidazole (hypoxic fraction) was significantly correlated with the fraction positive for pAKT (r_s_=0.51, p=0.04) (Figure 4). These results suggest that pAKT is activated by hypoxia in an EGFR-independent way in these tumors.

### pAKT is induced by hypoxia in vitro, and is important for cell survival under hypoxia

**Figure 5:**
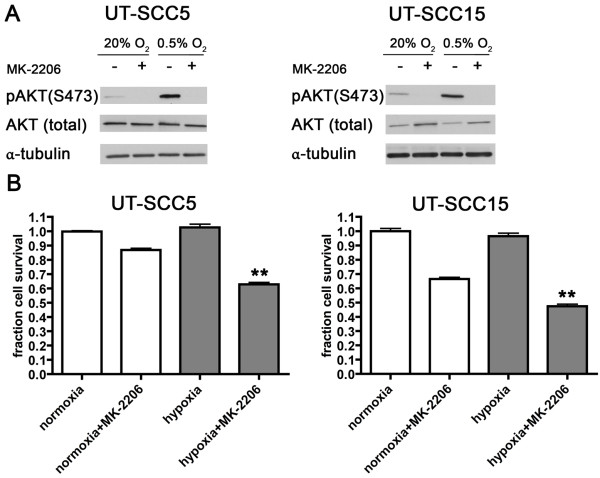
**Effect of hypoxia and MK-2206 on pAKT levels and cell survival *in vitro*. A**) Expression of (p)AKT after treatment with MK-2206 under normoxic and hypoxic (1h, 0.5% O_2_) conditions in UT-SCC5 and UT-SCC15. **B**) Cell survival after treatment with MK-2206 under normoxic and hypoxic (72h, 0.5% O_2_) conditions in UT-SCC5 and UT-SCC15. **: p<0.01 compared to control.

To confirm that hypoxia itself is an activating factor for AKT *in vivo*, cells of two HNSCC lines were cultured under hypoxic conditions (0.5% O_2_). One hour after hypoxia, pAKT was indeed increased in both cell lines (Figure 5A). As pAKT plays an important role in cell survival, these results suggest that hypoxic cells could be specifically targeted by pAKT-inhibition. To test the hypothesis that hypoxic cells are more sensitive to pAKT-inhibition, both normoxic and hypoxic (0.5% O_2_) cells were treated with the specific pAKT-inhibitor MK-2206 and cell survival was assessed. MK-2206 treatment decreased pAKT expression efficiently under normoxic and hypoxic conditions (Figure 5A). In both HNSCC cell lines hypoxia itself did not cause a decrease in cell survival (Figure 5B). Under normoxic conditions, MK-2206 had a small, but non-significant, effect on cell survival. However, when hypoxic cells were treated with MK-2206 this resulted in a significant decrease in cell survival (p<0.01). These results indicate that activation of AKT by hypoxia is important for cell survival under hypoxic conditions and can be specifically targeted by MK-2206.

## Discussion

In recent years, a variety of newly developed targeted anti-cancer therapies have successfully been combined with classic therapies, including the combination of EGFR-inhibition and radiotherapy in HNSCC [2]. However, these new intensified combinations also induce increased toxicity and (acquired) resistance [3,13]. The *a priori* selection of patients that will respond is thus paramount, and research has focused on the identification of resistance mechanisms using different *in vitro* models. However, the complex tumor microenvironment can also be a source of therapy resistance. Hypoxia can have a great impact on the behavior of a tumor cell and also on the response to treatment [14]. Therefore, it is important to determine which proteins are influenced by hypoxia, because these proteins could be potential targets to reduce therapy resistance to a variety of treatments, including radiotherapy and EGFR-inhibition.

In the current study, we found that the expression of EGFR and HER2 *in vitro* was correlated with the *in vivo* expression, which indicates that the expression of these tyrosine kinase receptors is largely an intrinsic feature of a tumor cell. The *in vitro* expression of activated EGFR (pEGFR) was also highly correlated with the *in vivo* expression, while the levels of pHER2 *in vivo* were clearly higher and varied more in the xenografts than *in vitro*. These observations suggest that activation of EGFR is possibly more genetically determined, e.g. by mutations present in the different tumor lines, while the activation of HER2 is more determined by factors in the tumor microenvironment that are not present in standard 2D cell culture. Also, in head and neck cancer patients a strong correlation between EGFR and pEGFR levels has been observed [15]. This correlation supports our observation that the extend of activated EGFR is predominantly influenced by EGFR overexpression and not by other stimuli in the microenvironment.

In contrast to the tyrosine kinase receptors, no *in vitro-in vivo* correlation was observed for the activated kinases pAKT, pERK1/2 or pSTAT3, indicating that the expression of these activated kinases is influenced by factors in the tumor microenvironment. Here, we show that hypoxia is an activating stimulus for AKT *in vivo*. This hypoxia-induced increase in pAKT cannot be explained by hypoxia-induced activation of EGFR as is observed in different *in vitro* models [16,17]. EGFR expression was namely predominantly present in oxygenated areas and there was a relatively low overlap between EGFR and pAKT expression *in vivo*. Besides these observations in our preclinical models, mismatch in EGFR-pAKT expression and the presence of pAKT in hypoxic regions is also observed in HNSCC patient samples [8]. EGFR-independent upregulation of pAKT by hypoxia has also been observed in lung cancer cells, whereby activation of AKT was induced via the IGF1R/PI3K/AKT pathway [18]. Also oxidative stress, which can occur during reoxygenation, has been shown to activate AKT in HNSCC cells [19]. Hypoxia-induced, EGFR-independent, activation of AKT could thus be an important resistance mechanism in HNSCC patients treated with EGFR-inhibition and radiotherapy. Although EGFR is the most commonly overexpressed tyrosine kinase receptor in head and neck cancer, also other receptors are overexpressed like HER2, HER3, and IL-6 receptor [20,21], which could possibly play a role in hypoxia-induced activation of AKT. However, we focused on our major finding that activation of AKT is a characteristic of hypoxic cells in HNSCC and therefore a potential target to specifically kill hypoxic cells. Extensive crosstalk between different growth factor receptors, such as EGFR and MET, has been reported [22]. These growth factor receptors activate similar pathways, which means that cells that overexpress multiple growth factor receptors can sustain survival signaling even if one of the receptors is blocked [23]. This is exemplified by the study of Erjala et al., which also used a panel of UT-SCC cell lines, that showed that EGFR or pEGFR levels were not correlated, but pHER2 and HER3 levels were correlated with sensitivity to EGFR-inhibition [4]. Also downstream signaling molecules like pAKT en pERK1/2 were not correlated with sensitivity for EGFR-inhibition. In our cell lines, we did also not observe that overexpression of pEGFR was consistently linked to overexpression of pSTAT3, pAKT or pERK1/2. Therefore, it is more important to determine activation of the common downstream pathway, which is responsible for cell survival, as this will be a more attractive target to overcome treatment resistance than targeting one specific growth factor receptor. In the HNSCC tumor lines studied, we indeed show that pAKT-inhibition decreases cell survival in hypoxic cells, but not in normoxic cells. Hypoxic cells are resistant to a variety of treatment regimens, including radiotherapy [9], and as pAKT signaling is an important cell survival pathway [24], targeting of pAKT in hypoxic tumors could be a promising way to significantly improve patient outcome. Additionally, multiple animal studies have shown that MK-2206 also inhibits pAKT *in vivo* and reduces tumor growth [25-27]. Moreover, Knowles et al. showed that MK-2206 not only reduced primary tumor size in an orthotopic HNSCC model, but also inhibited HNSCC migration *in vitro* and reduced the number of lymph node metastases *in vivo*[25]. Although the effect of AKT-inhibition on HNSCC migration could explain the reduced metastases formation in this study, it is also known that hypoxia can induce a metastatic phenotype [28]. Killing hypoxic cells via pAKT-inhibition, as we show in this study, could thus potentially reduce not only tumor growth, but also the metastatic potential of the tumor. AKT is also a highly druggable target in the clinic since multiple specific AKT inhibitors, including the inhibitor we used in our study, are already tested in phase I/II clinical trials and are generally well tolerated [29].

Although our data show that the microenvironment can induce the expression of activated kinases and that therefore expression levels in tumors do not correspond with cells *in vitro*, they do not explain why we see very little variation in total expression between the tumors. A possible reason for this is the technique we used to determine expression levels. Western blot analysis determines the total expression in all tumor cells together. However, using immunohistochemistry we observed that the level of expression of the different proteins varied widely between cells in the tumors under the influence of e.g. hypoxia and this spatial information is totally lost by western blot analysis. Thus, possibly by determining the expression in all cells together, these differences between individual cells level out and result in an ‘average’ level of expression, which differs relatively little between tumors as we observed in this study. One of the main advantages of immunohistochemistry is the possibility to analyze specifically tumor cells. Although we used tumors that had a large fraction of viable tumor cells and a very low amount of stromal cells, we cannot exclude the possibility that the presence of small amounts of normal cells affected our results.

## Conclusion

Our data indicate that the expression of (p)EGFR and HER2 is largely an intrinsic feature of a tumor cell, while the *in vivo* expression of activated kinases of important cell signaling pathways can be substantially affected by the tumor microenvironment. Moreover, we show that AKT is activated by hypoxia both *in vivo* and *in vitro* and that hypoxic cells are more sensitive to AKT-inhibition. As hypoxic tumors are resistant to a variety of treatment regimens, AKT might thus be a promising druggable target in hypoxic tumors. Further research is warranted to confirm our hypothesis that anti-cancer treatment can be improved by specifically targeting hypoxic cells with pAKT-inhibition and in which way this finding can be optimally used to improve patient outcome in the future.

## Competing interests

The authors declare that they have no competing interests.

## Authors’ contributions

HS designed and coordinated the project, performed the animal experiments and western blot analyses and drafted the manuscript. JHK, AJK, and JB obtained funding for this project and participated in its design and coordination and drafted the manuscript. PNS helped with the statistical analyses and interpretation of the data and revised the manuscript. DLW and MI participated in the design and interpretation of the data. SJW and MMV designed and performed the cell culture experiments. RG provided the cell lines and revised the manuscript. All authors read and approved the final manuscript.

## Pre-publication history

The pre-publication history for this paper can be accessed here:

http://www.biomedcentral.com/1471-2407/12/463/prepub

## Supplementary Material

Additional file 1 **Figure S1.***In vitro* and *in vivo* expression of (p)HER2, (p)STAT3 and (p)ERK1/2 in 6 HNSCC lines. Cell lines were both cultured as cell lines (*in vitro*) and grown as xenograft tumors (*in vivo*) and expression levels were determined with western blot. A) *In vitro* expression of (p)HER2, (p)STAT2 and (p)ERK1/2. B) *In vivo* expression of (p)HER2, (p)STAT2 and (p)ERK1/2. Number of harvested tumors ranged from 2 to 4 per cell line. Click here for file

Additional file 2 **Figure S2.** Enlarged detail of Figure 3. Expression of EGFR, pAKT and hypoxia in a tumor of UT-SCC5. Left picture: EGFR (red), pAKT (green), vessels (blue). Right picture: EGFR (red), hypoxia (green), vessels (blue). Magnification: 100X. Click here for file
