# Supplementary figures and images for: Activation of AKT by hypoxia: a potential target for hypoxic tumors of the head and neck
Source: BMC Cancer. 2012 Oct 10;12:463. doi: 10.1186/1471-2407-12-463 (PMC3517352; doi:10.1186/1471-2407-12-463)

# Supplemental Figure 1

**A**

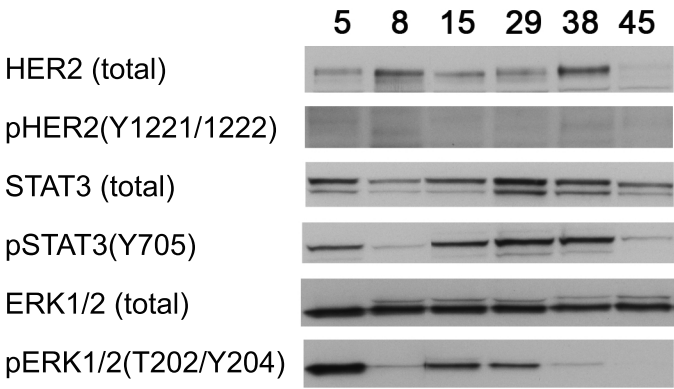

**B**

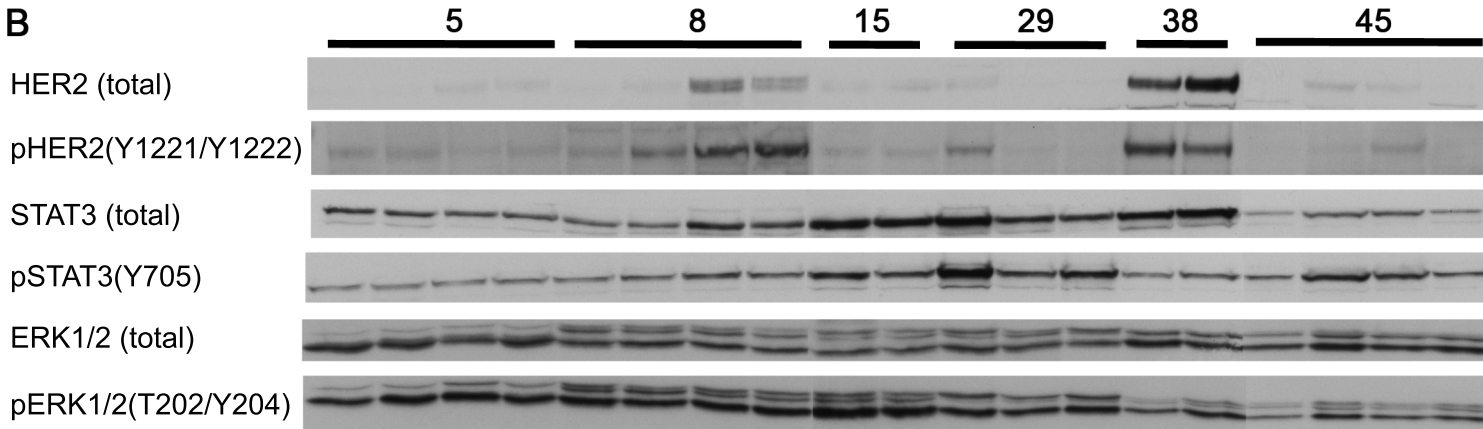

Supplement: Additional file 1 — Figure S1.In vitro and in vivo expression of (p)HER2, (p)STAT3 and (p)ERK1/2 in 6 HNSCC lines. Cell lines were both cultured as cell lines (in vitro) and grown as xenograft tumors (in vivo) and expression levels were determined with western blot. A) In vitro expression of (p)HER2, (p)STAT2 and (p)ERK1/2. B) In vivo expression of (p)HER2, (p)STAT2 and (p)ERK1/2. Number of harvested tumors ranged from 2 to 4 per cell line. [file 1471-2407-12-463-S1.pdf]

Supplemental Figure 2

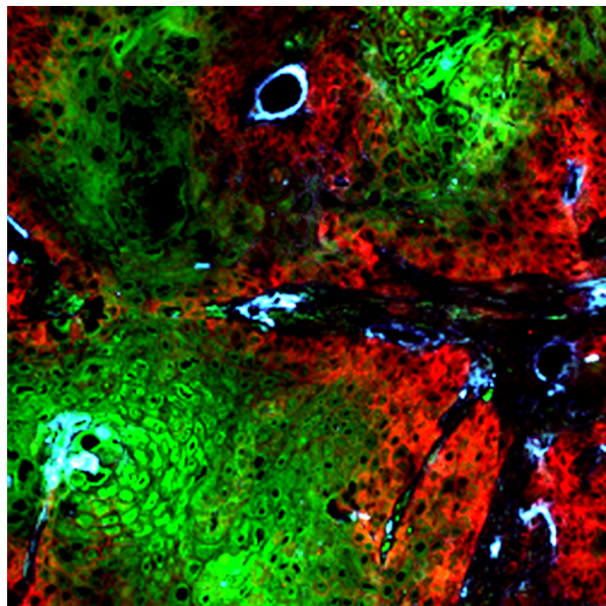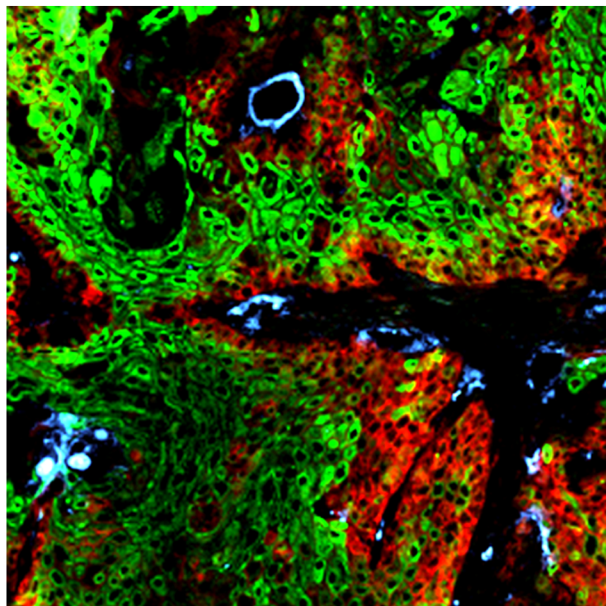

Supplement: Additional file 2 — Figure S2. Enlarged detail of Figure 3. Expression of EGFR, pAKT and hypoxia in a tumor of UT-SCC5. Left picture: EGFR (red), pAKT (green), vessels (blue). Right picture: EGFR (red), hypoxia (green), vessels (blue). Magnification: 100X. [file 1471-2407-12-463-S2.pdf]
